# Caregiver perspectives on barriers and facilitators to pediatric intestinal failure quality of life

**DOI:** 10.1016/j.intf.2025.100302

**Published:** 2025-08-06

**Authors:** Vikram K. Raghu, Lisa Lakkis, Claire Josey, Flor de Abril Cameron, Daniela Gattini, Beverly Kosmach-Park, Janel Hanmer

**Affiliations:** a University of Pittsburgh School of Medicine, Pittsburgh, PA, USA; b UPMC Children’s Hospital of Pittsburgh, Pittsburgh, PA, USA; c Institute of Health Policy, Management and Evaluation, University of Toronto, Toronto, Ontario, Canada; d Hospital for Sick Children, University of Toronto, Toronto, Ontario, Canada

**Keywords:** Intestinal rehabilitation, Short bowel syndrome, Pediatrics

## Abstract

**Background:**

Caregivers of children with intestinal failure provide unique perspectives on their child’s quality of life. This study explores the caregiver perception of barriers and facilitators affecting quality of life in children with intestinal failure and examines how caregiver quality of life may be affected.

**Material and methods:**

Semi-structured interviews were conducted with caregivers of children with intestinal failure and transcribed verbatim. An initial codebook was inductively developed by one coder and subsequently reviewed by two additional coders, who applied the codes to all transcripts. Thematic analysis was performed, and a conceptual framework was generated.

**Results:**

Interviews were conducted with 10 caregivers (9 biological mothers, 1 biological father). Quality of life domains may be grouped into 3 main categories: mental and psychological well-being, daily routine, and activities. Barriers to quality of life within these categories included differences from peers and siblings, mental health struggles, medical complications, procedures and hospitalizations, traditional school settings, swimming, and travel. Facilitators focused on adapting to these differences through support systems at home and school along with the medical care expertise gained over time. Caregivers faced significant impacts on their own quality of life in the areas of mental health, finances, and social activities.

**Conclusion:**

Caregivers offer valuable insight into the barriers and facilitators of their child’s QOL within the context of intestinal failure. Multidisciplinary interventions aimed at supporting families as they adapt to their own unique lifestyle may significantly enhance quality of life for both children and caregivers.

## Introduction

Children with intestinal failure (IF) are now expected to survive through childhood into adulthood. With advancements in intestinal rehabilitation, IF results in lower mortality than in previous decades [1-3]. Thus, attention has shifted from a focus solely on maximizing quantity of life to jointly maximizing quality of life (QOL).

Quality of life attempts to capture the subjective differences experienced by individuals that may provide more nuance than survival alone. Several studies have examined QOL through the lens of caregivers [4-6]. Many of these studies utilize existing questionnaires to measure QOL, yet these questionnaires often lack evidence of validity in children with intestinal failure. Moreover, summarizing questionnaire results may miss many of the individualized nuances of intestinal failure.

Key to the individual considerations in intestinal failure QOL is the role of the family. Often, parents and other caregivers play a critical role in managing this care. This provides them with unique insight into how intestinal failure affects child QOL. Conversely, child disease has a major impact on the entire family with current knowledge limited to primarily survey-based studies [7,8]. One single-center study used caregiver focus groups and found significant expressed burden of caregivers [4].

Semi-structured interviews provide a unique framework for capturing rich qualitative data from individual caregivers regarding their experiences caring for children with intestinal failure. Our recent work validating a specific health utility measure identified three primary domains that may be used to measure QOL: happiness, daily routine, and activities [9]. In the current study, we aim to examine how caregiver responses to questions about QOL may shed light on the barriers and facilitators to improving QOL in IF. Secondarily, we aim to understand how caregivers view their own QOL.

## Material and methods

We performed a secondary analysis of semi-structured interviews of caregivers of children with intestinal failure to define barriers and facilitators of child QOL and describe the interaction with caregiver QOL. Semi-structured interviews were chosen as the appropriate methodology for this study to allow for diverse viewpoints to be individually explored in a heterogeneous population. This study was approved by the local Institutional Review Board (STUDY21100136). Verbal consent was obtained from all participants. The original content analysis was previously published [9].

Caregivers with a child receiving parenteral nutrition followed at the local intestinal rehabilitation clinic were recruited. Caregivers were excluded if they could not participate in an English-language interview. Half-hour semi-structured interviews were conducted via Zoom with a single interviewer trained in performing qualitative interviews (FC). Each interview followed an interview guide that included questions about the caregiver’s perception of the child’s QOL and general QOL domains that are important to children with intestinal failure, and how the child’s health affects the caregiver’s QOL. Interviews were auto-transcribed by Zoom and then edited for accuracy. Interviews were conducted until thematic saturation was achieved with a minimum plan for nine interviews based on the minimum necessary to reach thematic saturation [10].

After performing our previous deductive analysis, this analysis used an inductive approach to identify themes related to the topics of barriers and facilitators of QOL and the caregiver’s perception of their own QOL. Interviews were coded using NVivo v14 (Lumivero, Denver, CO, USA). Two coders (LL and CJ) reviewed all transcripts and coded each one. Discrepancies in coding that could not be resolved by the two coders were brought to a third coder for a final determination (VR).

## Results

As previously reported, from an initial twenty-three caregivers recruited to participate, ten caregivers successfully completed interviews (9 biological mothers, 1 biological father). Diagnoses included short bowel syndrome (n = 8), congenital enteropathy (n = 1), and intestinal dysmotility (n = 1). There was an equal distribution of child sex based on caregiver report during the interview.

**Fig. 1.**
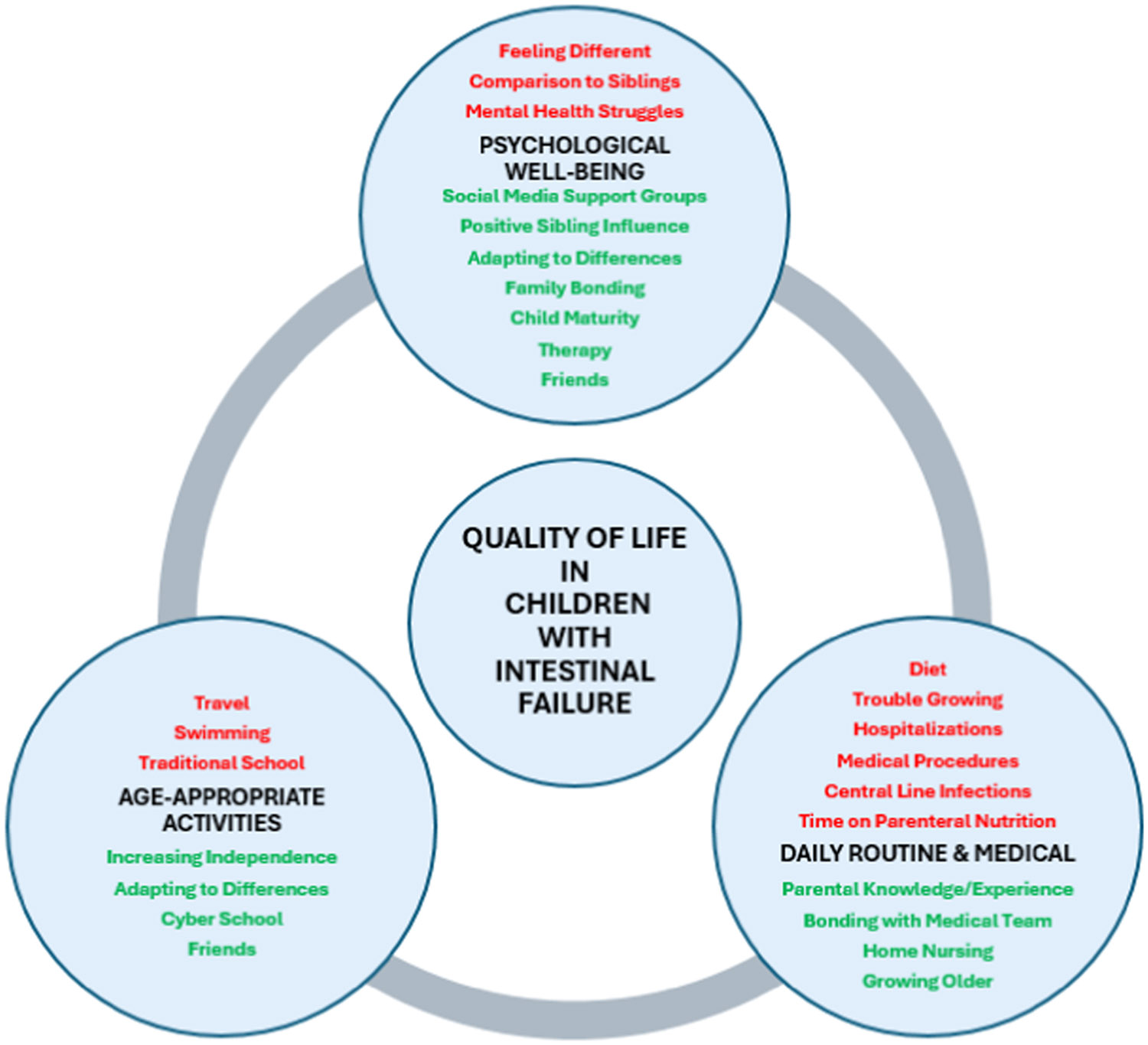
Conceptual framework of barriers and facilitators of intestinal failure quality of life. Circles represent the three domains identified by caregivers as the most important in their child’s quality of life. Barriers (red) and facilitators (green) within each domain are listed.

**Fig. 2.**
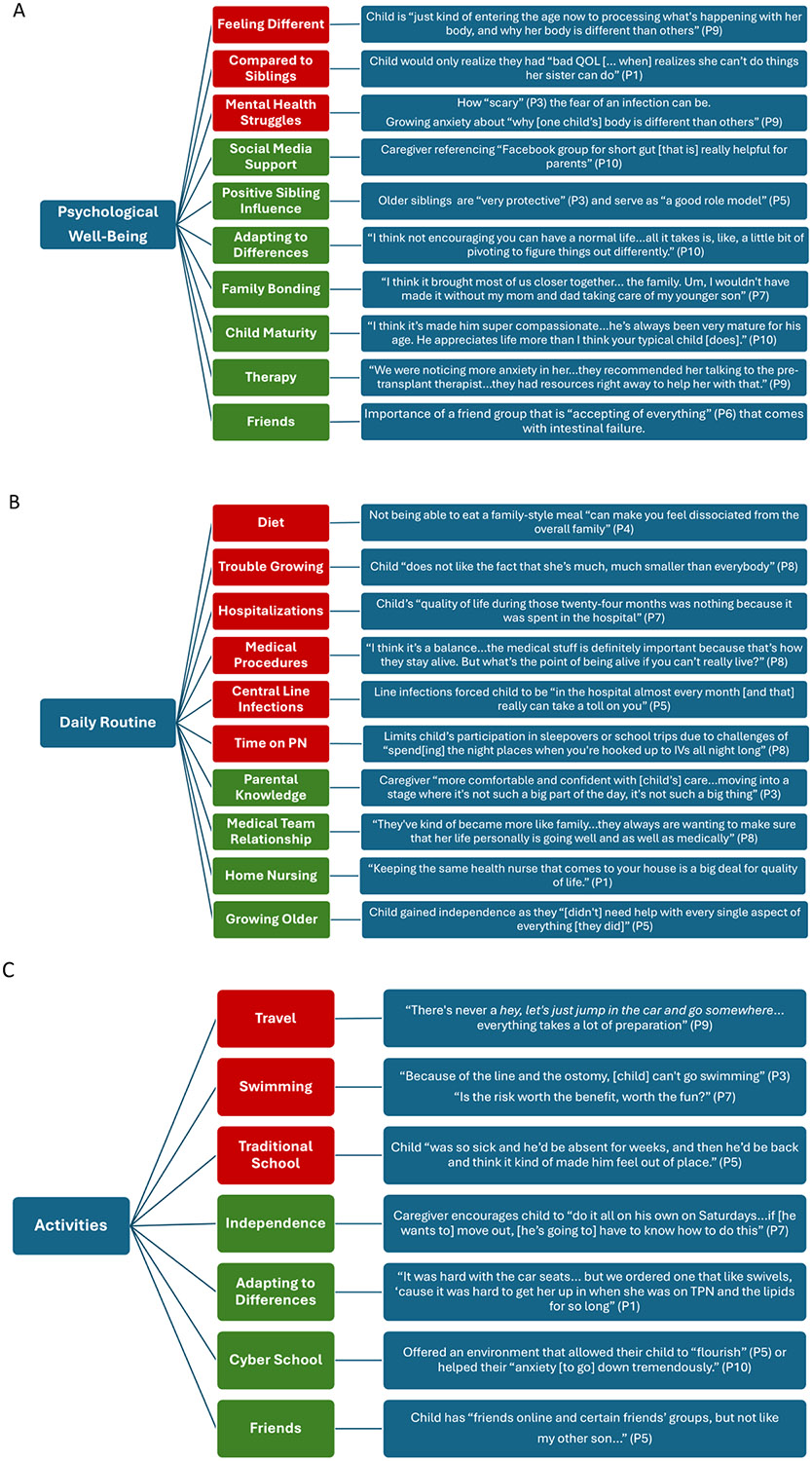
Quotes from caregivers supporting each barrier (red) and facilitator (green) within the quality-of-life domains of Psychological Well-Being (A), Daily Routine (B), and Activities (C).

Fig. 1 shows a thematic map of the barriers and facilitators to QOL for children with intestinal failure as noted by their caregivers within the framework of three primary domains: mental/psychological well-being, daily routine/medical care, and age-appropriate activities. The following sections will highlight barriers and facilitators in each domain with relevant quotes followed by a section on caregiver QOL (Fig. 2).

### Psychological well-being

#### Barriers:

The mental/psychological well-being domain included barriers identified by caregivers that highlighted differences between their child and other children, including peers and siblings. One caregiver noted that their child was “just kind of entering the age now to processing what’s happening with her body, and why her body is different than others” (P9). Another said their child would only realize they had “bad QOL [… when] realizes she can’t do things her sister can do” (P1). More formal mental health concerns often focused on anxiety towards both medical and non-medical aspects of care, ranging from how “scary” (P3) the fear of an infection can be to the growing anxiety over time about “why [one child’s] body is different than others” (P9).

#### Facilitators:

Although caregivers identified these differences and anxiety-provoking experiences, they also acknowledged the important role of a strong support system that ranged from caregivers and siblings to peers. Older siblings contributed by being “very protective” (P3), aiding with medical care, and serving as “a good role model” (P5). Multiple caregivers stressed the importance of a friend group that is “accepting of everything” (P6) that comes with having intestinal failure. Family included members beyond the nuclear family, as several caregivers spoke of grandparents, aunts, and stepparents that played a major role in facilitating improved QOL. Families themselves could find an additional layer of support through support groups, especially via social media, with one caregiver referencing a “Facebook group for short gut [that is] really helpful for parents” (P10).

Across all QOL domains, one caregiver voiced a strong feeling that the goal needs to look different. “I think not encouraging you can have a normal life. I think that’s one thing that we tried so hard in the beginning to just do everything we always expected we would do. And all it takes is, like, a little bit of pivoting to figure things out differently” (P10). This theme of accepting and adapting to differences spans all three aspects of QOL as an important facilitator.

### Daily and medical routines

#### Barriers:

Many barriers in attaining the caregivers’ perspective of a good QOL centered around how IF affected the child’s daily routine. Time was cited as an important factor in all aspects of their child’s life, particularly in the absence of time for typical childhood and family activities, because that time was needed to tend to the child’s medical routine. One caregiver noted that after an involved surgery, their child’s “quality of life during those twenty-four months was nothing because it was spent in the hospital” (P7). Another caregiver described how line infections forced their child to be “in the hospital almost every month [and that] really can take a toll on you” (P5). Time on parenteral nutrition was initially seen as a barrier to development when children were very young. As they approached school age with opportunities for increased activities and interactions both at home and in the community, it became increasingly important to schedule infusions on a nightly routine to improve their ability to participate in activities. Although nightly infusions may benefit the routine, it is not the perfect solution in that it limits the child’s ability to participate in sleepovers or school trips due to the challenges of “spend[ing] the night places when you’re hooked up to IVs all night long” (P8). From the clinical perspective, two caregivers commented about their child’s height with one pointing out that their child “does not like the fact that she’s much, much smaller than everybody” (P8). Dietary restrictions played a significant role in the eyes of most parents with one stating that not being able to eat a family-style meal “can make you feel dissociated from the overall family” (P4).

#### Facilitators:

Over time, caregivers believed that the child’s medical routine had a lesser impact on their daily routine as their child’s medical concerns became less daunting due to increased knowledge and experience. One caregiver stated that they became “more comfortable and confident with [their child’s] care, kind of moving into a stage where it’s not such a big part of the day, it’s not such a big thing” (P3). Children also gained independence as they “[didn’t] need help with every single aspect of everything [they did]” (P5). Nearly every caregiver mentioned that they felt like part of the medical team, with one caregiver outlining the unexpected nightly routine they now follow: “I never expected to push my son’s intestines back into his body every night whenever I do a bag change after I have hooked up an IV” (P10). Others focused on how they manage “all of [their child’s] medical needs, which is a 24/7 job” (P9). While family members engage in many aspects of medical care, the medical team members take on a different role as well with one mother stating, “they’ve kind of became more like family […] they always are wanting to make sure that her life personally is going well and as well as medically” (P8). The home nurse played an equally important role in QOL with one caregiver stressing that “keeping the same health nurse that comes to your house is a big deal for quality of life” (P1).

### Age-appropriate activities

#### Barriers:

Many childhood activities posed a challenge for children with IF. QOL issues were commonly highlighted in traditional school settings, where caregivers “had to figure out a kid-friendly way to explain [their child’s condition]” (P1), contend with peer issues such as bullying that would make their child “a little bit scared to go to school” (P5), or missing school time because of routine medical needs, as in having an ostomy that “needs emptied at least once an hour” (P10). Children also had to contend with illnesses, with one caregiver noting that their child “was so sick and he’d be absent for weeks, and then he’d be back and think it kind of made him feel out of place” (P5). Of the typical childhood activities, families most commonly discussed swimming. Many described how “because of the line and the ostomy, [their child] can’t go swimming” (P3), but as one caregiver put it, “is the risk worth the benefit, worth the fun?” (P7). While some families have “tried to basically just erase it” (P10) from the conversation, others have chosen “to kind of find a safe way for [their child] to do it” (P8) through dry suits or limiting to familiar locations such as their private pool. Traveling with parenteral nutrition was another limitation frequently cited by caregivers. For some, the limitation was related to staying “close enough to a hospital to get there in [the required] time frame and that they have what he needs when we get there” (P3). Others have made the extra effort to travel because “making memories as a family is just extremely important to [them]” (P9). However, that caregiver made it clear that “there’s never a *hey, let’s just jump in the car and go somewhere* […] Everything takes a lot of preparation” (P9). Families provided examples ranging from limits on how much parenteral nutrition they could carry without special requests to the home infusion company to packing enough supplies in case of an emergency.

#### Facilitators:

Despite these challenges, families realized that finding ways for their child to participate in typical activities improved their QOL. Many children elected to attend cyber school, with one caregiver noting that since making that transition their child was “a lot more healthier and not going to the hospital as much” (P2). For children with numerous daily medical and physical needs such as emptying an ostomy, enteral feedings, and infusions, cyber school offered an environment that allowed them to “flourish” (P5) or helped their “anxiety [to go] down tremendously” (P10). One caregiver noted that with the transfer to cyber school, “it is harder to make friends” (P10), but several found alternative social engagements to fill that gap. Another caregiver described their child with IF as having “friends online and certain friends’ groups, but not like my other son,” in reference to the differences in the ways they engage with friends. Many families focused on the importance of their children starting to take on more responsibility for their daily medical care as a key step in attaining independence. One caregiver encourages their child to “do it all on his own on Saturdays,” stating to him that “if [he wants to] move out, [he’s going to] have to know how to do this” (P7).

### Caregiver QOL

#### Barriers:

Caregiver QOL is impacted by many barriers. Among these, the most frequently cited are mental stressors and time constraints that affect both work and social life. When caring for younger children, caregivers agreed that the condition was “a lot more stressful I think on the parents than on the kids” (P1). One caregiver described the constant worrying about their child’s health and the frequent trips to the hospital as “a miserable hell” (P2). During that time, each caregiver would “put [themselves] on the back burner, left [their] health go” with a subsequent plan “to get back to where [they] should be again” (P2). Caregivers completely removed themselves from other aspects of society, with one stating, “For a long time, I didn’t have a life. I didn’t work. I didn’t socialize. […] I won’t say I lost a lot of friends, but we’re not as close as we were” (P7). Financial stressors ranged from one caregiver voluntarily “sell [ing] his business and [becoming their child’s] full-time caregiver” (P9) to “more money out of a parent’s pocket” (P2) due to the demands of seeking care for their child. Four caregivers specifically mentioned one or both caregivers losing or quitting their job due to the demands of providing care.

#### Facilitators:

Caregivers noted that improved QOL was associated with the amount of support they received in providing their child’s care. Although parents provided the majority of the care, they voiced the importance of having another family member trained both as a backup and as support to provide “a little bit of a break, you know, just for a night out to dinner to still be a married couple is important” (P10). One family leaned heavily on their church community for support, stating that, “they all love her and treat her like any other little girl […] at our church” (P9). While no families mentioned specifically using the hospital for respite, one caregiver said, “when we’re in the hospital that’s some of the best sleep I get” (P7).

When asked specifically how their child’s health affects their life, one caregiver said, “Does it affect my QOL? Yes, it does. But sometimes it’s good, sometimes it’s bad” (P4). In fact, another caregiver focused on that positive aspect, stating, “He’s made mine better. […] He has made me see things different, like as far as probably being more appreciative of certain things” (P5) One caregiver said simply about her child that “her life is my life” (P8) and described how their child’s health was the “the biggest factor in [their own] quality of life” (P8).

## Discussion

Caregivers play a critical role in the lives of children with IF. and their perspectives provide unique insights into their own and their children’s lived experiences. This qualitative study identified barriers and facilitators within three primary dimensions of QOL for caregivers caring for children with IF. The overall theme among the facilitators was finding ways to adapt to the differences posed by IF care while the barriers may be described as how differences from the norm (daily routine, peers, activities) are exacerbated.

In using these data to improve QOL for children with IF, interventions to impact or improve participation in the typical activities of childhood should be prioritized and strategized, such as adaptations for travel, swimming, and school participation. Through the Covid-19 pandemic, remote and cyber school models became more widespread with improving structures [11,12]. While traditional in-person schools provide more opportunities for socialization, it seems that a more flexible schooling model may be beneficial for these children when deemed appropriate. In fact, work during the pandemic suggested that remote learning does lead to increases in communication between parents and teachers as well as family time [11,12]. On the other hand, in-person schooling may come with learning supports or other services that cannot be recreated virtually. Consequently, caregivers should be aware of educational models available in their local area, and in collaboration with their child, make an informed decision regarding in-person school participation.

In terms of swimming, discussions often focus on the serious and potentially fatal risks of infection. Swimming is an important part of normal child development and socialization [13-16]. Although the infectious risks are real and significant [17,18], the absolute risk attributable to swimming has not been quantified nor compared with other childhood activities. Moreover, it is clear from our interviews that children often still participate in swimming even if the medical team discourages it. If families choose to pursue swimming, interventions such as dry suits may attenuate some of the risk. This highlights the need for shared discussions over activities that a family deems critical to QOL. Members of the multidisciplinary intestinal rehabilitation team such as physical therapy and occupational therapy may play a pivotal role in these discussions both to support water-based activities and to provide alternatives for physical activity. This aligns with recent guidelines underscoring the importance of motor development for these children [19].

Travel with a child receiving parenteral nutrition can be very challenging for families to coordinate, often limiting the length of the trip and the potential locations to remain close to an intestinal rehabilitation center [17]. Yet, some families have discovered ways to work with home infusion companies and their care teams to make more extensive family travel possible. Societal guidelines have outlined recommendations to ease the burden of traveling with parenteral nutrition and central venous catheters [17].

Together with activities like swimming and alternative schooling options, these conversations may deserve a more proactive approach early in the intestinal failure journey, especially as families consider how to achieve some degree of normalcy. Our data suggest that intestinal rehabilitation teams should carefully consider how to approach these conversations while acknowledging the caregiver’s perception of their child’s QOL. Caregivers should be counseled on how to optimally adapt to the differences they perceive and guided to achieve optimal QOL. A multidisciplinary team approach, particularly with the support of team members who can address the psychosocial elements of care (e.g., psychologists, social workers, and child life specialists) can help the caregiver and child identify goals and collaboratively achieve them.

Our proposed conceptual framework of QOL for the child with IF suggests how the various barriers and facilitators may impact the specific QOL domains considered the most important by our families. As longitudinal assessment of QOL continues in IF, these data may produce several hypotheses as to how to improve QOL for these children through specific causal pathways that target important domains. For example, finding ways to support swimming for children with central lines may specifically improve their activities domain. This can potentially produce measurable benefits to QOL.

Caregiver QOL seems to be directly tied to the caregiver’s perception of the child’s QOL. At times, it was difficult to distinguish whether caregivers were reflecting on their perception of their own QOL or their child’s, especially when discussing the benefits of home nursing, family support, or just the experience they gained over time. This suggests that caregivers asked to proxy report on child QOL may struggle due to the interdependence with their own QOL, which has been suggested by many pediatric quality-of-life studies across illnesses [20]. This interdependence may play a crucial role in considering the benefits of intestinal failure treatments as the ultimate change in QOL may magnify with the effect it produces on the entire family. These spillover effects, as they are often called, should be the focus of future quality-of-life work.

Our study adds to the growing body of literature suggesting that caring for a child with IF can negatively impact caregiver QOL [4,6,21,22]. Previous work has highlighted the higher rates of caregiver depression and anxiety, limitations on social life, and significant financial hardships [4,6,21-23]. These studies have suggested numerous interventions for improving caregiver QOL, including support groups, screening for and intervening in the case of mental health conditions, and addressing financial burdens [4,6,21,22]. Many of these concerns highlight the key role that mental health experts and social workers play in the intestinal rehabilitation team. Societal guidelines have included these team members as a recommended but not required member of the intestinal rehabilitation team. We strongly feel that a psychologist and a social worker can be vital parts of supporting many of the challenges described by caregivers for both themselves and their children.

Support of caregiver mental health likely has a direct impact on child mental health and quality of life. Child mental health, primarily anxiety around medical care, had a critical role in defining the psychological well-being of the child. As such, it is imperative that both child and caregiver mental health are addressed by the intestinal rehabilitation team for the sake of the child. Failure to do so may have far-reaching negative impacts, particularly to the detriment of child well-being.

Our study is limited by the geographic distribution of families from a single center. The child’s perspective of their QOL was not assessed in this study. These issues are being addressed by an ongoing multicenter qualitative study of adolescents and young adults with pediatric-onset intestinal failure.

## Conclusion

Caregivers of children with intestinal failure have a unique perspective on QOL that demonstrates the interdependence between their own QOL and their child’s. Improving child normalcy across all domains, especially in terms of travel, school, and activities such as swimming may have direct impacts on QOL for both children and their families. Significant stressors, both psychological and financial, continue to impact caregivers and highlight the critical role of mental health professionals and social workers in the intestinal rehabilitation team.

## References

[1] Gattini D, Roberts AJ, Wales PW, Beath SV, Evans HM, Hind J, Mercer D, Wong T, Yap J, Belza C, Huysentruyt K, Avitzur Y. Trends in pediatric intestinal failure: a multicenter, multinational study. J Pediatr 2021;237:16–23. 10.1016/j.jpeds.2021.06.025. e4. 34153281

[2] Raghu VK, Belaid S, Gutierrez S, Holzer P, Orris S, Rothenberger S, Presel T, Ackerman K, Alissa F, King D, Woo Baidal J, Rudolph JA, Bond G, Mazariegos GV, Horslen SP, Smith KJ. Social and financial costs of neonatal intestinal failure. JAMA Netw Open 2025;8(2):e2459548. 10.1001/jamanetworkopen.2024.59548. 40009383 PMC11866025

[3] Raghu VK, Leraas HJ, Samoylova M, Park C, Rothenberger SD, Sudan D, Avitzur Y. Predictors of 1-year enteral autonomy in children with intestinal failure: a descriptive retrospective cohort study. JPEN J Parent Enter Nutr 2023;47(8):1047–55. 10.1002/jpen.2557. PMC10843595 37573479

[4] Belza C, Patterson C, Ghent E, Avitzur Y, Ungar WJ, Fehlings D, Stremler R, Wales PW. "Line care governs our entire world": Understanding the experience of caregivers of children with intestinal failure receiving long-term parenteral nutrition. JPEN J Parent Enter Nutr 2022;46(7):1602–13. 10.1002/jpen.2337. 35088428

[5] Neumann ML, Allen JY, Kakani S, Ladner A, Rauen MH, Weaver MS, Mercer DF. A beautiful struggle: parent-perceived impact of short bowel syndrome on child and family wellbeing. J Pedia Surg 2022;57(9):149–57. 10.1016/j.jpedsurg.2021.09.039. 34702565

[6] Neam VC, Faino A, O’Hara M, Wendel D, Horslen SP, Javid PJ. Prospective evaluation of the family’s health-related quality of life in pediatric intestinal failure. JPEN J Parent Enter Nutr 2022;46(3):652–9. 10.1002/jpen.2212. 34170551

[7] Belza C, Avitzur Y, Ungar WJ, Stremler R, Fehlings D, Wales PW. Stress, anxiety, depression, and health-related quality of life in caregivers of children with intestinal failure receiving parenteral nutrition: a cross-sectional survey study. JPEN J Parent Enter Nutr 2023;47(3):342–53. 10.1002/jpen.2461. 36336350

[8] Belza C, Ungar WJ, Avitzur Y, Stremler R, Fehlings D, Wales PW. Carrying the burden: informal care requirements by caregivers of children with intestinal failure receiving home parenteral nutrition. J Pediatr 2022;250:75–82. 10.1016/j.jpeds.2022.05.049. e3. 35660493

[9] Raghu VK, Lakkis L, de Abril Cameron F, Valdes DG, Kosmach-Park B, Hanmer J. Applicability of the Child Health Utility instrument to measure health utility in children with intestinal failure: a qualitative study of caregivers. Intest Fail 2024;2:100024. 10.1016/j.intf.2024.100024 PMC12851196 41648084

[10] Hennink M, Kaiser BN. Sample sizes for saturation in qualitative research: A systematic review of empirical tests. Soc Sci Med 2022;292:114523. 10.1016/j.socscimed.2021.114523. 34785096

[11] Steed EA, Leech N, Phan N, Benzel E. Early childhood educators’ provision of remote learning during COVID-19. Early Child Res Q 2022;60:307–18. 10.1016/j.ecresq.2022.03.003. 35340352 PMC8934718

[12] Roy AK, Breaux R, Sciberras E, Patel P, Ferrara E, Shroff DM, Cash AR, Dvorsky MR, Langberg JM, Quach J, Melvin G, Jackson A, Becker SP. A preliminary examination of key strategies, challenges, and benefits of remote learning expressed by parents during the COVID-19 pandemic. Sch Psychol 2022;37(2):147–59. 10.1037/spq0000465. 35266770

[13] Yu Y, Xia L, Yan H, Lu Y. Effects of 8 weeks parent-accompanied swimming on physical capacity and intelligence in preschool children. Front Public Health 2024;12:1410707. 10.3389/fpubh.2024.1410707. 38883199 PMC11176541

[14] Kano H, Ebara T, Matsuki T, Tamada H, Yamada Y, Kato S, Kaneko K, Matsuzaki K, Sato H, Minato K, Sugiura-Ogasawara M, Saitoh S, Kamijima M. Effect of swimming initiation period and continuation frequency on motor competence development in children aged up to 3 years: the Japan environment and children’s study. BMC Sports Sci Med Rehabil 2024;16(1):192. 10.1186/s13102-024-00980-9. 39285428 PMC11406841

[15] Jia M, Hu F, Yang D. Effects of different exercise modalities on pediatric and adolescent populations with developmental disorders: a network meta-analysis of randomized controlled trials. Eur J Pediatr 2024;184(1):18. 10.1007/s00431-024-05858-z. 39546034

[16] Leo I, Leone S, Dicataldo R, Vivenzio C, Cavallin N, Taglioni C, Roch M. A non-randomized pilot study on the benefits of baby swimming on motor development. Int J Environ Res Public Health 2022;19(15). 10.3390/ijerph19159262. PMC9368508 35954617

[17] Wendel D, Mezoff EA, Raghu VK, Kinberg S, Soden J, Avitzur Y, Rudolph JA, Gniadek M, Cohran VC, Venick RS, Cole CR. Management of central venous access in children with intestinal failure: a position paper from the NASPGHAN intestinal rehabilitation special interest group. J Pediatr Gastroenterol Nutr 2021;72(3):474–86. 10.1097/MPG.0000000000003036. 33399327 PMC8260029

[18] Miller J, Dalton MK, Duggan C, Lam S, Iglesias J, Jaksic T, Gura KM. Going with the flow or swimming against the tide: should children with central venous catheters swim? Nutr Clin Pract 2014;29(1):97–109. 10.1177/0884533613515931. 24347530 PMC4632199

[19] So S, Patterson C, Palsa T, Mears J, Bondi BC, Dempster S, Belza C. Neurodevelopmental, cognitive and motor function: Practice recommendations in pediatric intestinal failure and transplantation- position statement of the International Intestinal Rehabilitation and Transplant Association allied health committee. Intest Fail 2025;5:100062. 10.1016/j.intf.2025.100062. PMC12851274 41648854

[20] Thomas S, Ryan NP, Byrne LK, Hendrieckx C, White V. Psychological distress among parents of children with chronic health conditions and its association with unmet supportive care needs and children’s quality of life. J Pediatr Psychol 2024;49(1):45–55. 10.1093/jpepsy/jsad074. 37840456 PMC10799716

[21] van Oers HA, Haverman L, Olieman JF, Neelis EG, Jonkers-Schuitema CF, Grootenhuis MA, Tabbers MM. Health-related quality of life, anxiety, depression and distress of mothers and fathers of children on Home parenteral nutrition. Clin Nutr 2019;38(4):1905–12. 10.1016/j.clnu.2018.06.981. 30017244

[22] Winkler MF, Smith CE. Clinical, social, and economic impacts of home parenteral nutrition dependence in short bowel syndrome. JPEN J Parent Enter Nutr 2014;38(1):32S–7S. 10.1177/0148607113517717. 24418898

[23] Samuelsson M, Wennick A. An exploratory study of the everyday life of swedish children on home parenteral nutrition and their families. J Pedia Nurs 2020;52:e84–9. 10.1016/j.pedn.2020.01.010. 32044196

